# Factors associated with hepatitis B vaccine series completion in a randomized trial for injection drug users reached through syringe exchange programs in three US cities

**DOI:** 10.1186/1471-2458-14-820

**Published:** 2014-08-09

**Authors:** Sarah Bowman, Lauretta E Grau, Merrill Singer, Greg Scott, Robert Heimer

**Affiliations:** Yale University School of Public Health, New Haven, CT USA; Department of Emergency Medicine, Rhode Island Hospital, Providence, RI USA; Department of Epidemiology of Microbial Diseases and the Center for Interdisciplinary Research on AIDS, Yale University School of Public Health, New Haven, CT USA; Hispanic Health Council, Hartford, CT USA; Department of Anthropology, University of Connecticut, Storrs, CT USA; Department of Sociology, DePaul University, Chicago, IL USA

**Keywords:** Hepatitis B, Vaccination, Syringe exchange programs, Injection drug users

## Abstract

**Background:**

Hepatitis B virus (HBV) is a vaccine preventable infection yet vaccination rates are low among injection drug users (IDUs) despite the high risk of infection and longstanding recommendations to promote vaccination. We sought to improve vaccination rates by reaching IDUs through syringe exchange programs (SEPs) in three U.S. cities.

**Methods:**

**I**DUs were randomized in a trial comparing the standard HBV vaccination schedule (0, 1, and 6 months) to an accelerated schedule (0, 1, and 2 months) and participation data were analyzed to identify determinants of completion of the three-dose vaccine series. Independent variables explored included sociodemographics, injection and syringe access behaviors, assessment of health beliefs, HBV-associated knowledge, and personal health status.

**Results:**

Covariates associated with completion of the three-dose vaccine series were accelerated vaccine schedule (aOR 1.92, 95% CI 1.34, 2.58, p = <0.001), older age (aOR 1.05, 95% CI 1.03, 1.07, p = <0.001), and poorer self-rated health score (aOR 1.26, 95% CI 1.05, 1.5, p = 0.02). Completion was less likely for those getting syringes from SEP customers than for SEP customers (OR 0.33, 95% CI 0.19, 0.58, p = <0.001).

**Conclusions:**

SEPs should offer hepatitis vaccination in a manner that minimizes time between first and last visits by accelerating the dosing schedule. Public health interventions should target younger, less healthy, and non-SEP customer participants. Other health interventions at SEPs may benefit from similar approaches that reach out beyond regular SEP customers.

## Background

The availability since 1982 of a safe and effective vaccine against the hepatitis B virus (HBV) has dramatically reduced the annual incidence of acute HBV infections from an estimated 13.8 cases per 100,000 population in 1987 to an estimated 1.5 cases per 100,000 population in 2007 [1, 2]. Nevertheless, one risk group with continued high incidence is injection drug users (IDUs) [3–5]. Despite consistent recommendation from the Advisory Committee on Immunization Practice to target IDUs, many remain unvaccinated [5–8]. Studies of HBV vaccine programs delivered either by referral to health clinics or on-site at syringe exchange programs (SEPs) have concluded that offering convenient locations and modest financial incentives, including contingency management approaches, greatly increased vaccine uptake and completion of the three-dose series [4, 9–12]. A third approach to improving completion of the series is accelerating the schedule to shorten the usual six-month schedule. An accelerated version of the vaccine series administered over the course of two months has been shown to be similarly effective [13, 14].

Drawing on this prior research to maximize vaccine uptake among active IDUs we conducted the Hepatitis Vaccine Study (HVS) that included all three components: using syringe exchanges to find eligible high risk individuals, paying them to participate, and comparing the standard six-month and an accelerated two-month HBV vaccination schedule. The comparison was conducted as a randomized trial in at SEPs in three cities -- Hartford and Bridgeport in Connecticut and Chicago in Illinois. Participants were offered a modest monetary incentive for each dose received. A previous analysis concerned the cost-effectiveness of the two strategies [15]. Here we report our analysis of factors associated with non-completion of the vaccine series. We hypothesized that the longer interval of the standard regimen, less frequent use of the SEP to obtain syringes, low hepatitis B knowledge, and low self-efficacy would be associated with non-completion of the vaccine series.

## Methods

The research protocol was approved by the IRBs at Yale University, DePaul University (Chicago) and the Hispanic Health Council (Hartford). Participants received $10-15 for each study visit that they completed (i.e., up to $75 for the five visits).

### Study sample and procedures

An in-depth description of the study sample and procedures has been published elsewhere [15, 16]. Participants were enrolled between May 2003 and March 2006 at SEP locations in the three cities. Individuals were eligible to receive the vaccine if they could demonstrate evidence of having injected within the past 30 days (injection stigmata), were 18 years of age or older, were screened for and found susceptible to HBV, and were deemed able to provide informed consent. Oral consent for screening and subsequent participation based on HBV vaccination and infection status was obtained orally prior to screening.

To determine eligibility for vaccination, participants were screened by serological testing for antibodies to core and surface antigens (HBcAb and HBsAb, respectively) and for surface antigen (HBsAg). Individuals who tested negative for all three tests were informed that they were susceptible to HBV infection and invited to receive vaccination through the study. Individuals who tested HBcAb-positive, regardless of the test results for HBsAb were informed that they had been previously infected. Individuals who tested HBsAb-positive but negative on the other tests were informed that they had been previously successfully vaccinated. Individuals who tested HBsAg-positive, regardless of either of the other two HBV results, were informed that they were likely to be actively infected, instructed when they returned for their results to seek medical care, and advised on how to avoid transmitting the virus to others.

**Figure 1:**
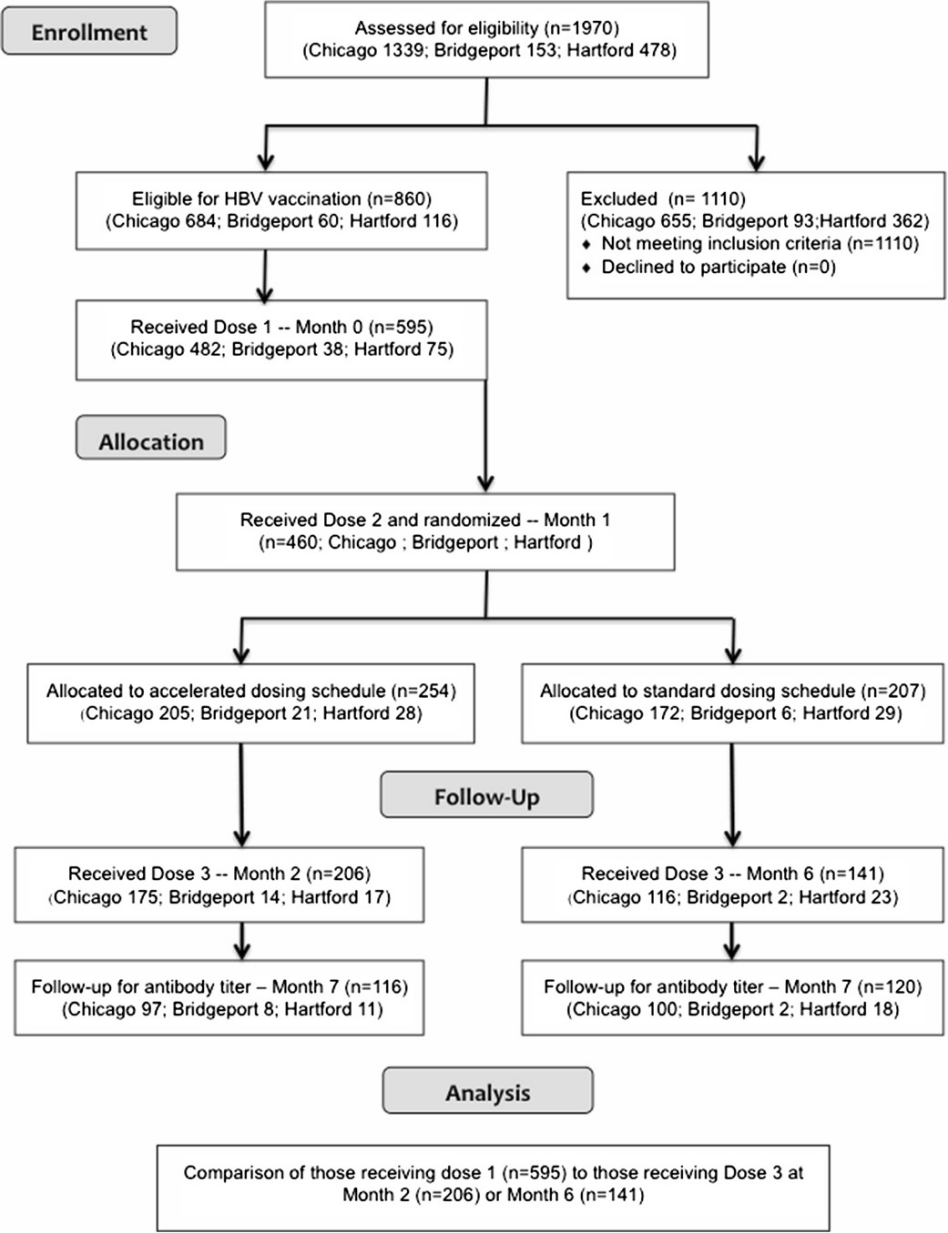
**CONSORT schematic for the standard and accelerated arms of the HVS study.**

Those whose serologic tests results indicated that they were susceptible to HBV infection were administered the first vaccine dose when they returned for their test results and then instructed to return for the second dose one month later. A random numbers assignment log, maintained at each study site, was used to assign participants to either the standard (0, 1, and 6 months) or accelerated (0, 1, and 2 months) schedule at the Dose 2 visit. All participants were invited to return for a final visit seven months after their first dose to assess acquisition of protective immunity, and in Chicago a fourth dose was given to those in the accelerated arm. The recruitment, intervention, and analysis scheme is shown in Figure 1.

### Data collection

The outcome of interest was administration of the final vaccine dose (Dose 3) among those receiving Dose 1. A set of covariates was included to test our hypotheses and identify confounders. These included treatment group, SEP usage variables, city of enrolment, sociodemographic variables, current health status variables, injection practices, and history of either engaging in or receiving commercial sex work services. Hepatitis knowledge and psychosocial factors were also assessed. The hepatitis knowledge instrument was an adaptation of the National Institute on Drug Abuse Risk Behavior Assessment questionnaire and included 22 questions using a True/False/Don’t Know response format to assess knowledge of routes of infection, disease detection and treatment, and prevention options [17, 18]. The psychosocial instrument, developed specifically for this study and grounded in Protection Motivation Theory, contained 21 items that assessed the six constructs in the theoretical model [19, 20]. These included respondents’ perceptions about (1) personal vulnerability to HBV infection, (2) disease severity for HBV infection, (3) response efficacy of specific prevention behaviors (e.g., using SEPs, refusing to share injection equipment) in reducing risk of HBV infection, (4) self-efficacy for engaging in specific prevention behaviors, (5) social peers’ approval of the respondent engaging in HBV risk reduction behaviors (i.e., social outcome expectancy), and (6) the importance of social approval from their peers (i.e., social outcome value). Items were phrased as an “I- statements” and used a 5-point Likert response format ranging from Strongly Agree to Strongly Disagree. The number of items included in each sub-scale ranged from one (vulnerability and severity subscales) to seven (response efficacy) items. Inter-item correlations (Cronbach’s alpha) for the multiple-item subscales were calculated and ranged from 0.62 to 0.86. Data obtained from these items were converted to z-scores, and the mean score for each subscale was calculated. A series of questions were included concerning motives for participation that included remaining healthy, protecting the health of others, participating in research, and receiving compensation.

### Analytic methods

Statistical analyses were conducted using SAS version 9.1. Logistic regression was used to calculate bivariate associations between all co-variates and the outcome. Covariates that were significantly associated with vaccine completion at the p ≤ 0.10 level in the bivariate analyses were subsequently entered into a multivariate logistic regression model with backwards elimination of any covariate that did not remain significant at the p < 0.05 level or did not change other coefficients by >10%.

We also explored several areas of potential collinearity. To test the hypothesis that those earning more money were more likely to have had a recent medical visit and less likely to use SEPs [21, 22], chi-square tests were used to test for associations between income and doctor visits during the past year and income and SEP utilization. The laws governing syringe exchange varied with there being no limit to the number of syringes that could be received at each visit in Chicago in contrast to a cap of 30 syringes that could be received on a one-for-one basis in Hartford and Bridgeport. Therefore, to test if there was an interaction between participants’ main source of syringes and their city of enrollment, an interaction term was created and entered into a multivariate logistic regression including the interaction term and main effects with receiving Dose 3 as the outcome.

## Results and discussion

Of the 860 individuals who screened susceptible to HBV infection, 595 (69.2%) returned for the Dose 1 visit and were enrolled in the vaccine study. A total of 460 participants (77.3% of those receiving Dose 1) returned for their second dose. Of the 271 randomized to the standard group, 141 (52.0% of Dose 1 recipients) returned for Dose 3, and 120 (44.4% of Dose 1 recipients) returned for the follow-up visit. Among the 324 randomized to the accelerated arm, 206 (63.6% of Dose 1 recipients) returned for Dose 3, and 116 (35.8% of Dose 1 recipients) returned for the follow-up visit.

Table 1 presents the results of the bivariate analyses. As hypothesized, completion of the vaccine series was significantly higher for participants in the accelerated arm versus the standard arm, for those receiving most of their syringes directly from the SEP where they were enrolled in the study as opposed to another source, and for direct SEP customers than for people whose main source of syringes in the past 30 days had been secondary exchange (i.e., a direct SEP customer provided the participant with SEP syringes). In addition, completion rates were higher among those offered vaccination at the Chicago SEP, among non-Hispanic Blacks versus Hispanics, among women versus men, among those unemployed versus employed, and among those of older age, and poorer self-rated health status. The results did not support the hypotheses that greater HBV knowledge or self-efficacy to be vaccinated would be significantly associated with completion. In addition, perceived motivations for participating were not significantly associated with completion of the vaccination series. Further analysis revealed no interactions between city and main source of syringes over the past three months, between income and most recent medical visit within the past year, or between income and SEP utilization (data not shown).

**Table 1:** **Unadjusted correlates of dose 3 completion**

	N*	uOR	95% CI		p-value
Accelerated treatment group	595	1.61	1.16	2.24	0.01
City	595				
Chicago		Reference			
Hartford		0.75	0.46	1.22	0.25
Bridgeport		0.48	0.24	0.93	0.03
Race	588				
Non-Hispanic Black/African American		Reference			
White		0.74	0.49	1.13	0.15
Hispanic		0.63	0.43	0.94	0.02
*Excluded Native American/Alaskan Native, Other, Refused (n = 7)*					
Women	594	1.45	1.00	2.11	0.05
Age (continuous, range 18–68)	595	1.05	1.03	1.07	<.01
Education	581				
Less Than high school		Reference			
High school or graduate equivalency degree		0.94	0.63	1.39	0.74
Some College or College graduate		1.15	0.76	1.75	0.51
*Excluded missing other (14) and vocation schools (11)*					
Employment (Full- or part-time)	578	0.66	0.44	1.01	0.05
Average monthly income					
$0-300		Reference			
$301-556		0.87	0.52	1.45	0.58
$557-1,000		1.10	0.70	1.73	0.68
$1,001-9,000		0.76	0.47	1.23	0.26
Not homeless	577	0.93	0.66	1.31	0.68
*Excluded missing (n = 14), and don’t know (n = 4)*					
Pay for housing	582	1.43	1.03	1.99	0.04
Self-reported health status (Likert 1 = excellent, 5 = poor)	578	1.21	1.02	1.42	0.03
*Excluded missing (13) and don’t know (4)*					
Told by health care worker HCV pos	576	0.97	0.59	1.60	0.90
*Excluded missing (15) and don’t know/unsure (4)*					
Doctors visit during past year	582	1.06	0.74	1.51	0.80
Customer of local syringe exchange	539	0.98	0.64	1.49	0.91
Main source of syringes, prior 30 days	558				
SEP direct		Reference			
Pharmacy		0.45	0.19	1.05	0.06
SEP customer		0.37	0.22	0.64	<0.01
Diabetic, someone else, at place you shoot		0.79	0.47	1.34	0.38
*Excluded missing (37)*					
Most syringes from this exchange, past 3 months	462	1.59	1.07	2.37	0.02
EVER referred by SEP to healthcare/drug tx/social service	575	1.29	0.28	2.00	0.26
*Excluded missing (17) don’t know (3)*					
Average # of shots from a syringe (range 1–75)	557	0.98	0.93	1.02	0.31
Total injections, prior 30 days (range 1–540)	521	1.00	0.99	1.00	0.84
*Excluded does not apply (n = 25)*					
Used a needle someone else had used at least, prior 30 days	553	1.73	0.90	3.33	0.10
Someone paid you w/ drugs or money for sex	580	1.21	0.81	1.80	0.36
You paid someone with drugs or money for sex	575	0.83	0.55	1.25	0.38
Language of interview	582				
English		Reference			
Spanish		0.57	0.30	1.10	0.09
Language spoken most often	578				
English		Reference			
Spanish		0.76	0.45	1.27	0.30
*Excluded missing and other (n = 3)*					
Hepatitis Knowledge	585	2.78	0.45	17.10	0.27
Vulnerability	581	1.01	0.85	1.19	0.94
Severity	581	1.10	0.93	1.30	0.25
Response efficacy	580	1.01	0.85	1.19	0.94
Self-efficacy	581	0.93	0.79	1.10	0.39
Social outcome expectancy	580	1.04	0.88	1.23	0.64
Social outcome value	581	1.05	0.89	1.24	0.57

*The number of people answering each question is included, numbers may not equal 595 due to missing data.

All covariates that were significant at the p ≤ 0.10 level in the bivariate analyses were entered into a multivariate model (Table 2). Four variables remained significantly associated with completion of the vaccine series such that those who completed the series were more likely: (1) to have been randomized to the accelerated treatment group, (2) to be direct SEP customers rather than to report using secondary exchange as their main source of syringes in the past thirty days, (3) to be older, and (4) having poorer self-rated health status.

**Table 2:** Multivariate logistic regression of correlates associated with dose 3 completion (N = 430*)

	aOR	95% CI		p-value
Accelerated Treatment Group	1.92	1.34	2.58	<0.001
Age (continuous)	1.05	1.03	1.07	<0.001
Less healthy (Likert Scale 1–5)	1.26	1.05	1.5	0.01
Main source of syringes past 3 months				
Direct from SEP	Reference			
Direct from pharmacy	0.43	0.179	1.04	0.60
From SEP customer	0.33	0.19	0.58	<0.001
Other	0.68	0.39	1.18	0.17

*165 observations were excluded due to missing values for the outcome or explanatory variables.

### Methods

This analysis, designed to compare completion rates between two dosing schedules, was constructed on the hypothesis that completion rates would be higher among the accelerated group. A second manuscript comparing efficacy between those randomized to the accelerated versus the standard schedule is in preparation. As anticipated, vaccination completion was significantly more likely among participants in the accelerated treatment group, suggesting that shortening the vaccine schedule reduces the risk of encountering barriers to participation common among IDUs, which can include incarceration or competing health care and personal needs.

Low rates of completion of the three-dose HBV vaccine series have previously been documented among IDUs especially in, but not limited to, the United States [9, 23–25]. Accelerated HBV vaccine dosing schedules that have been implemented among hard-to-reach populations including active IDUs have been shown to increase completion rates [8, 26–29]. However, none of these prior studies has used SEPs as the access point for identifying and vaccinating active injectors. In our present study, despite offering financial incentives for vaccination at SEPs, only 58% of Dose 1 recipients completed the vaccine series. However, several studies have established a consensus that paying individuals do increase vaccination completion rates in drug injecting populations [10, 30, 31].

There has been discussion regarding whether or not a booster dose is necessary for the accelerated HBV vaccine series. Although benefits of a booster dose for the accelerated HBV vaccine schedule have been suggested, several studies have concluded that booster doses are neither necessary nor is maintenance of an antibody level ≥10 mIU/mL essential for protection because an anamnestic response has been detected up to 22 years post-vaccination [32–34]. Further research would be necessary to evaluate the feasibility of offering a 12-month booster dose at SEPs. Although this might not seem a priority in light of the above findings, the lower success rate for vaccination among people who inject drugs suggests that a booster might be appropriate in this population [24, 35–39].

Completion was slightly more likely among older participants, consistent with earlier findings of low vaccination rates among young injectors and among younger people in general [8, 11, 40]. Thus, special attention should be paid to recruiting and retaining younger participants, especially since they are less likely to have already been exposed to and infected with HBV (albeit increasingly more likely in the future to have been vaccinated against HBV infection in early childhood). Multifaceted interventions, targeting youth within their community deserve further consideration. A review of the literature on services and interventions for runaway and homeless youth concluded that interventions addressing the varied and interconnected needs of youth are more successful than those targeting one problem at a time [41, 42]. Further research is needed to specifically understand barriers to vaccine program completion among young injectors.

Participants with a poorer self-rated health score were more likely to complete the vaccine series. This runs counter to expectations from previous work that found competing needs served as a barrier to health care acquisition and preventive health care, particularly among vulnerable populations including homeless adults and IDUs [43–45]. This may, instead, be one example of the potential for collinearity in the variables in our dataset. Older individuals were more likely to report poorer health (data not shown) and were also more likely to complete the vaccine series.

Compared to direct SEP customers, a significantly lower completion rate was observed among people who engaged in secondary exchange. The benefit of providing health services at SEPs has been repeatedly demonstrated [3, 9, 12, 23, 46, 47], but this advantage may be of limited benefit to non-customers. Our findings suggest that recruitment of non-SEP customers should draw on peer networks in addition to more traditional recruitment strategies (e.g., outreach, posted fliers). SEP customers who distribute SEP syringes to non-customers should be encouraged to promote participation through their own social networks, word of mouth, or easy-to-distribute promotional cards. The underlying reasons that some IDUs do not go directly to SEPs were not assessed within the current study. Further research is needed to identify barriers to SEP utilization and alternative methods for targeting this hidden population of IDUs.

This study has a number of limitations. First, participants were predominantly direct or indirect SEP customers, so the findings have limited generalizability to the full IDU population in these communities. Second, as with any study that relies on self-reported behavioral data, this evaluation may be influenced by self-report and recall bias. Third, recruitment methods were intended to simulate what would be realistic and feasible for a typical SEP with limited resources and staffing to implement a vaccination intervention; the recruitment strategy may have resulted in a non-random sampling that may have introduced unmeasured bias into the study population. Fourth, lack of adequate specificity in some of the variables collected such as sources of income, a full medical history, and the precise nature of social relationships limited a full assessment of competing subsistence or health needs. Finally, enrolment was slow. It took three years to recruit sufficient participants to power the analysis. We have previously reported that an improved study design would have involved getting rid of the two-week waiting period for test result to identify those eligible for vaccination and giving the first dose of vaccine at enrolment [15]. This approach was also found to be most cost-effective.

## Conclusions

The findings from this evaluation lead us to conclude that IDUs can be encouraged to participate in preventative health promotion efforts through SEPs, although services at SEPs are most likely to reach only the direct SEP customers. Since most non-completers obtained their syringes from other SEP customers, drawing on peer networks to undertake a peer-driven intervention and maintain contact with non-customers may promote completion of the vaccine series [48–51]. Specific attention is also required to encourage completion among younger and/or healthier participants to obtain and complete HBV vaccination. Our findings furthermore suggest that SEPs offering hepatitis vaccination should consider minimizing the time between first and last visits, one strategy for which is offering the first HBV vaccination at the screening visit [15]. Finally, our results suggest that paying individuals to get vaccinated is only one of several programmatic requirements to insure maximum coverage.
